# Joint Contributions of Psychological Distress and Body Mass Index to Presenteeism: A Register-linked Follow-up Study Among Young and Early Midlife Municipal Employees

**DOI:** 10.1097/JOM.0000000000003800

**Published:** 2026-07-01

**Authors:** Mari-Liis Kalima, Anna C. Svärd, Jatta Valkonen, Kimmo Vänni, Tea Lallukka, Eira Roos

**Affiliations:** From the Department of Public Health, Faculty of Medicine, University of Helsinki, Helsinki, Finland (M.-L.K., A.C.S., J.V., T.L., E.R) and HAMK Tech – Häme University of Applied Sciences, Hämeenlinna, Finland (K.V.).

**Keywords:** cohort study, work ability, body weight, productivity loss, mental health

## Abstract

**Background:**

Psychological distress and overweight are increasingly prevalent public health concerns associated with reduced work ability and sickness absence, but their combined association with presenteeism is unknown.

**Methods:**

Survey data from the Helsinki Health Study (2017) of employees aged 19–39 (n = 3510) were linked with 12-month personnel register data. The association between psychological distress, body mass index and presenteeism was assessed using negative binomial adjusted for sociodemographic and work factors.

**Results:**

Presenteeism accounted for 4.4% of total working days. Psychological distress (18.2%) and overweight (41.9%) were each associated with higher presenteeism, with the strongest association for both combined (incidence rate ratio 2.91, 95% confidence interval 2.36–3.60), after adjustments.

**Conclusions:**

Psychological distress and overweight jointly increased presenteeism, highlighting the need for integrated workplace support to prevent productivity loss in this group with long work careers ahead.

## Abstract 2

Psychological distress and overweight/obesity are jointly associated with presenteeism among young and early midlife employees. As presenteeism may indicate early work ability decline, targeted mental and physical health support from employers and occupational healthcare is essential to prevent productivity loss in this group with long work careers ahead.

## Graphical abstract

**Figure:**
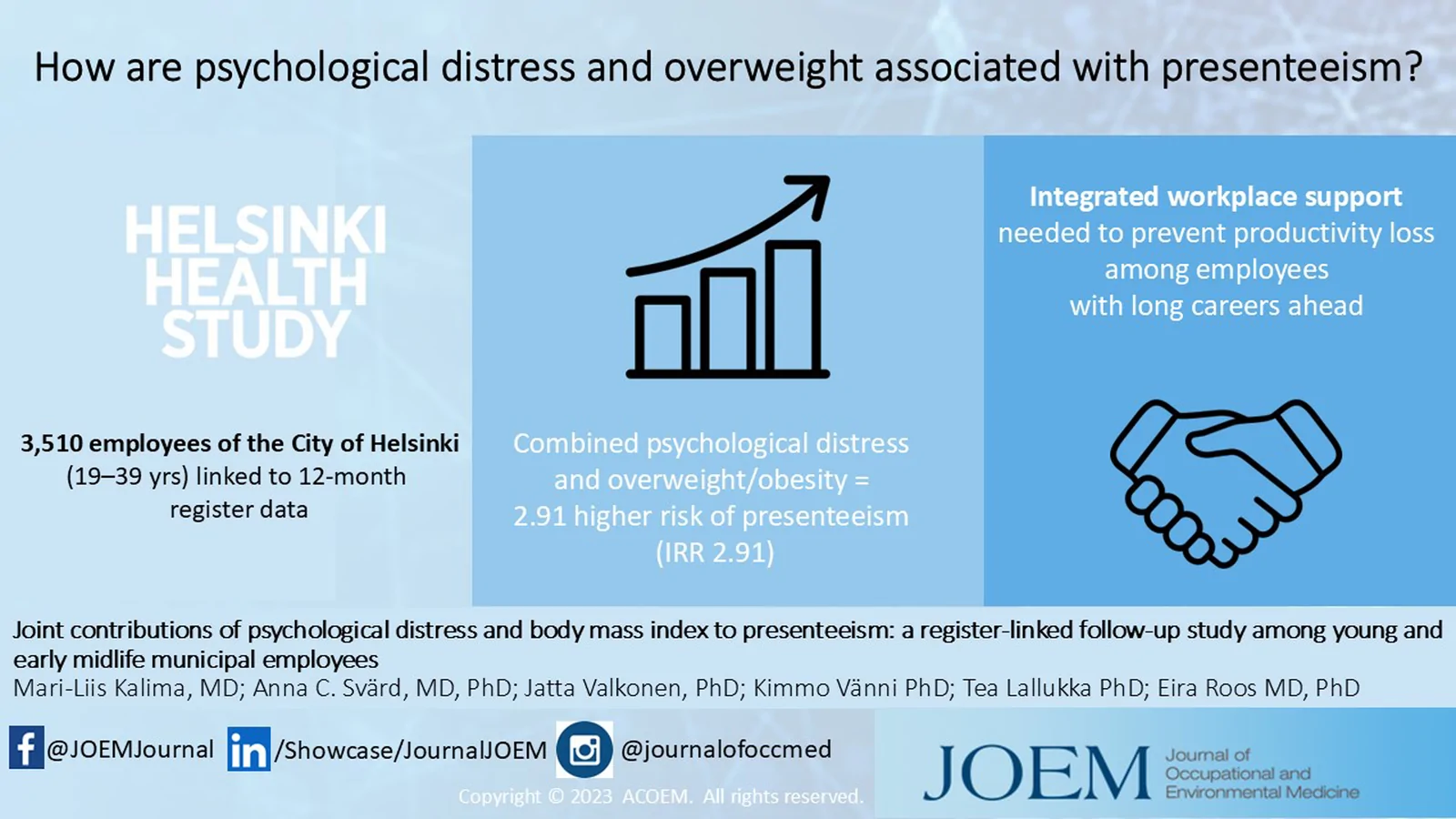

**LEARNING OUTCOMES**

- **Identify** the independent and joint associations of psychological distress and overweight with presenteeism among young and early midlife employees.
- **Explain** how coexisting mental and physical health burdens contribute to increased presenteeism, and potential early decline in work ability.
- **Apply** this new knowledge to support the development of targeted workplace interventions that address both mental well-being and weight management simultaneously.

Presenteeism, referring to underperforming owing to ill-health while at work,^1^ has become an increasingly recognized component of productivity loss, alongside sickness absence and permanent work disability. At the organizational level, presenteeism is associated with substantial economic costs, elevated healthcare expenditures, a higher risk of workplace accidents, and greater likelihood of errors.^1^ It is estimated that the overall costs associated with presenteeism surpass those of sickness absence.^2,3^ However, despite its economic and health implications, presenteeism remains notably less studied than sickness absence.

According to European Working conditions survey 2021, the annual presenteeism prevalence is known to vary by country, occupation, employment status and industry, ranging from 19% in Lithuania to 38% in Cyprus.^4^ Presenteeism is associated with both acute conditions (eg, flu, migraine, and back pain) and chronic diseases (eg, depression, diabetes, arthritis, and gastrointestinal disorders).^1,2^ However, employees facing unfavorable organizational factors such as job insecurity, strained working conditions, and high work demands also report more presenteeism.^5^ Employees who experience presenteeism report lower job satisfaction, reduced work engagement, and higher levels of emotional exhaustion.^1^ Presenteeism also predicts later poorer health, increased sickness absence, and may occur before and after sickness absence.^6–8^

Mental health problems and overweight/obesity (body mass index [BMI] ≥25 kg/m^2^) are prevalent public health concerns that contribute to productivity loss through presenteeism, sickness absence, and disability pension.^5,9,10^ Psychological distress and overweight/obesity are bidirectionally linked, with distress increasing obesity risk and obesity contributing to symptoms such as depression and anxiety.^11,12^ This relation may be driven by biological stress responses, emotional eating, and stigma related to higher body weight.^11,12^ Psychological distress, typically manifested as nonspecific stress, anxiety, or depression, is commonly assessed with screening tools designed to identify risk for common mental disorders.^13^ Among 20–39 years old Finnish adults, between 2018 and 2024 psychological distress has grown from 25% to 34%. ^14^ In earlier studies, psychological distress was associated with an increased likelihood of presenteeism.^5,15^ Moreover, overweight is increasingly common, affecting currently more than half of Finnish adults under 40 years,^16^ and it is associated with a higher prevalence of sickness absence.^17^ Nevertheless, evidence regarding the relation between overweight and presenteeism remains inconclusive. A Canadian national study reported only a weak association between obesity (BMI ≥30 kg/m^2^) and presenteeism among adults aged 20–69 years.^18^ In contrast, a Belgian survey of private and municipal employees aged 30–55 assessed presenteeism by self-reported frequency of working while ill.^19^ Associations between overweight and presenteeism were found only among men.^19^ In addition to independent associations, psychological distress and overweight are jointly associated with sickness absence. ^20^ To the best of our knowledge, the joint association of psychological distress and overweight with presenteeism has not been previously examined.

This study aimed to examine the joint association of psychological distress and overweight with presenteeism among young and midlife employees of the City of Helsinki, Finland. Based on previous research on perceived work ability, presenteeism, sickness absence, psychological distress, and overweight, we considered the potential roles of sociodemographic factors, working conditions, and health behaviors in these associations.^21–26^

## METHODS

### Participants

This study is part of the Helsinki Health Study, an ongoing cohort study on employees of the City of Helsinki that investigates social and work-related determinants of health and wellbeing.^27^ The City of Helsinki, the largest employer in Finland, has around 38,000 employees working in a wide range of occupations, from blue-collar to white-collar, with the majority in the education, social, and healthcare sectors. Phase 1 data were collected in 2017 via online and postal questionnaires among 19–39-year-old employees with at least a 50% employment contract for at least 4 months before the start of data collection (n = 11,459). The response rate was 51.5% (n = 5898) (Fig. 1). Among those who did not respond to the online or postal questionnaire, a shorter telephone interview was conducted (n = 787). Of the respondents, 80% were women, which reflects the gender composition of municipal employees in Finland.^27^ In all, 83% of the respondents consented to the register linkages. Nonresponse analysis indicated slight underrepresentation of those with lower socioeconomic position and long-term sickness absence, but the data broadly represent the target population.^27^

**FIGURE 1.**
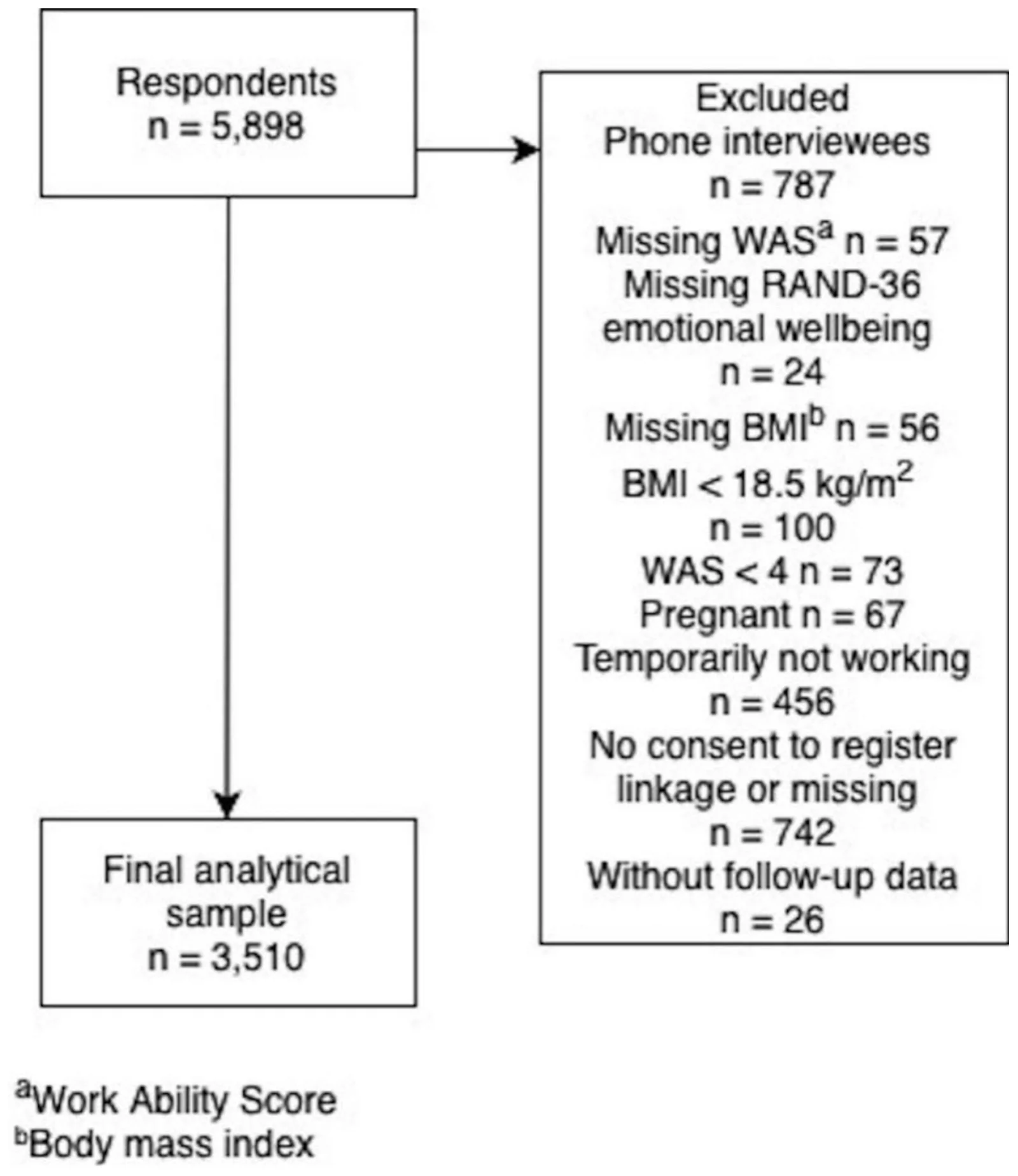
Flowchart of the selection of the Helsinki Health Study participants in phase 1 in 2017.

We excluded those who were interviewed by telephone (n = 787), as the phone interviews did not include the RAND-36 emotional wellbeing and other variables needed for this study. We excluded participants with missing data on Work Ability Score (WAS) (n = 57), the RAND-36 emotional wellbeing (n = 24), BMI (n = 56), and those having BMI under 18.5 kg/m^2^ (n = 100). Participants with BMI under 18.5 kg/m^2^ had slightly lower education level, were younger and were dominantly women. To reduce the risk of over expression of presenteeism, and as done in a previous study.^28^ participants reporting WAS under 4 points (2%, n = 73) were excluded. Respondents who reported pregnancy in an open form (1%, n = 67), who had not been working when returning the questionnaire (n = 456; on sick leave for over 6 months, on disability pension, receiving rehabilitation subsidy, temporarily studying, on parental leave, or unemployed), who did not consent to register linkage (n = 742), or who were without any follow-up data (n = 26) were excluded. The final analytical dataset included 2737 (78%) women and 773 (22%) men, (n = 3,510).

#### Body Mass Index

BMI was calculated using self-reported weight (kg) divided by self-reported height squared (m^2^). Participants were dichotomized according to World Health Organization (WHO) criteria into having a healthy weight (BMI 18.5–24.9 kg/m^2^) or overweight/obesity (BMI ≥25 kg/m^2^). Owing to a relatively small number of participants with obesity (BMI ≥30 kg/m^2^, n = 529), we combined those with overweight or obesity into the same group.

#### Psychological Distress

We measured psychological distress with the standardized and validated RAND-36 emotional wellbeing subscale, which consists of 5 questions reflecting mental well-being during the last 4 weeks.^29,30^ It reflects symptoms of anxiety, depression, and positive mood (“Have you been a very nervous person?,” “Have you felt so down in the dumps that nothing could cheer you up?,” “Have you felt calm and peaceful?,” “Have you felt downhearted and blue?,” and “Have you been a happy person?”). The questionnaire is widely used for assessing health-related quality of life and is identical with the emotional well-being scale of Short Form Survey-36 (mental health inventory-5), although there are slight coding differences between the 2.^29^ Transformed score was calculated ranging from 0 (worst possible health state) to 100 (best possible state). Psychological distress was dichotomized using a cutoff score of 60, based on previous studies that have been used with mental health inventory-5.^13,31^ This cut-off indicates moderate to severe psychological distress and has been shown to be optimal for identifying cases.^13,31^

#### Presenteeism and Sickness Absence Days

Data on job tenure and the number of sickness absence days were obtained from the personnel register of the City of Helsinki. The sickness absence follow-up began the day after the questionnaire was received and continued for 12 months or until the end of the participant’s employment contract, whichever occurred first. During the follow-up period, the City of Helsinki supervisors were authorized to grant sickness absence for up to 7 days without requiring a medical certificate.

Presenteeism days were counted with a tool developed in a Finnish study based on WAS.^28,32^ WAS is person’s estimation of their current work ability compared with their lifetime best on a scale from 0 to 10, where 10 indicated their best-ever work ability and 0 indicated full disability. We calculated the on-the-job productivity loss, that is, presenteeism days based on formula from a previous study using WAS-related discount factors.^28,32^ The discount factor for productivity loss percentages was 0%–26%; WAS 9–10 was interpreted as no productivity loss, while scores of 8, 7, 6, 5, and 4 corresponded to estimated productivity losses of 5%, 11%, 16%, 21%, and 26%, respectively. In the formula, the WAS-derived discount factor is multiplied by annual net working days after subtracting sickness absence days. Annual working days were set at 227, based on the Finnish average.^33^ We estimated net working days based on individual follow-up time and self-reported weekly working hours, using 38.25 hours as the standard for the Finnish public sector. Presenteeism days were calculated as the total over the 1-year follow-up period (mean follow-up 0.93 years).

### Covariates

The covariates were derived from the questionnaire. Gender included women and men. Age was classified into 3 categories: 19–29, 30–34, and 35–39 years.^34^ We dichotomized marital status into married/cohabiting and others (single, divorced, or widowed).^22^ Educational background was divided into 3 levels: lower or upper secondary school, bachelor’s degree, and master’s degree or higher.^22^ Physical working conditions were asked with 4 response options ranging from very light to very strenuous, and the answers were dichotomized into nonstrenuous (very/rather light) and strenuous (rather/very strenuous).^21,23^ Alcohol consumption was reported by types of alcohol drinks (beer/cider/wine/spirits), and the portions were summed together. Women consuming alcohol >7 portions/wk and men consuming >14 portions/wk were considered as risk-users, and those consuming less than these as nonrisk-users.^24,35^ Respondents with missing data were categorized separately (n = 133, 3.8%) owing to the high proportion of missing values for alcohol use. Participants’ smoking status was dichotomized into 2 categories: nonsmokers or former smokers, and current smokers.^25^ Physical activity was calculated from the respondent’s estimates of average weekly hours of leisure-time and commuting physical activity (ie, LTPA) per 4 intensity grades. We multiplied weekly hours by the metabolic equivalent (MET) values and then added the values together. LTPA was dichotomized according to the MET-hours into low (<20 MET-hours), and moderate or high (≥20 MET-hours).^26^ Participants who had missing information on marital status (n = 10), education (n = 7), physically strenuous work (n = 30), smoking (n = 4), physical activity (n = 39), working status (n = 8) were classified as single, having low education, having physically nonstrenuous work, nonsmoking, having high levels of LTPA, working full- or part-time, respectively, to reduce potential overestimation of associations. This is in line with previous procedures.^36–38^ Missing self-reported weekly working hours (n = 95) were treated as 38.25 hours/wk. Additional analyses excluding participants with missing working hours yielded essentially similar results (data not shown).

### Statistical Analysis

First, we used cross-tabulation to describe the distribution of the participants’ characteristics by gender (Table 1). Second, we calculated presenteeism days based on the WAS-related discount factor (Table 2). Presenteeism days were nonnormally distributed and therefore summarized using medians. Medians and interquartile ranges were compared by psychological distress, BMI, and joint exposure groups. The 4 joint exposure groups were defined based on psychological distress and BMI as follows: participants (1) without psychological distress and with a healthy weight, (2) with psychological distress and a healthy weight, (3) without psychological distress and with overweight, and (4) with psychological distress and overweight. Third, we used negative binomial regression to calculate the incidence and 95% confidence intervals (CIs) for presenteeism days per 10 person-years by exposure to psychological distress and overweight, both independently and by joint exposure groups (Fig. 2). Fourth, we calculated incidence rate ratios (IRRs) and their 95% CIs for presenteeism days by the joint exposure groups using negative binomial regression (Table 3). For the negative binomial regression analyses, we rounded decimal values to the nearest whole number to obtain integer count data. Participants without psychological distress and with a healthy weight served as the reference group. Model 1 was adjusted for gender and age, and Model 2 was additionally adjusted for marital status, education, and physical strenuousness of work. Model 3 was fully adjusted for gender, age, marital status, education, physical strenuousness of work, and health behaviors (alcohol consumption, smoking, and physical activity). There were no statistically significant gender interactions in the associations between the exposure groups and presenteeism (*P* = 0.813) and thus, we analyzed women and men together, adjusting the analyses for gender. Among participants, 12.9% had less than 12 months of follow-up. To account for varying follow-up times, the natural logarithm of follow-up time was included as an offset term in the models. Finally, we calculated the synergy index (*S*) to examine the synergistic interaction between psychological distress and overweight/obesity using the following equation: *S* = (IRR participants with psychological distress and overweight/obesity 2212 1)/([IRR participants with psychological distress and healthy weight − 1] + [IRR participants without psychological distress and with overweight/obesity − 1]).^39^ We used IRRs from Model 1 (adjusted for age and gender) and fully adjusted IRRs from Model 3. A synergy index >1 suggests a synergistic interaction.

**TABLE 1.** Characteristic of Young and Early Midlife Participants of the Helsinki Health Study Phase 1 in 2017

Characteristics at Phase 1	All	Gender	
		Women	Men
	n (%)	n (%)	n (%)
	3510	2737	773
Age			
19–29	1109 (31.6)	913 (82.3)	196 (17.7)
30–34	1162 (33.1)	910 (78.3)	252 (21.7)
35–39	1239 (35.3)	914 (73.8)	325 (26.2)
Marital status			
Married/cohabiting	2282 (65.0)	1718 (75.3)	564 (24.7)
Other	1228 (35.0)	1019 (83.0)	209 (17.0)
Education			
Upper secondary school	1161 (33.1)	838 (72.2)	323 (27.8)
Bachelor’s degree	1295 (36.9)	1060 (81.9)	235 (18.1)
Master’s degree or higher	1054 (30.0)	839 (79.6)	215 (20.4)
Physically strenuous work			
Nonstrenuous	2373 (67.6)	1798 (75.8)	575 (24.2)
Strenuous	1137 (32.4)	939 (82.6)	198 (17.4)
Alcohol consumption^a^			
Nonrisk-user	3136 (89.3)	2561 (78.3)	708 (21.7)
Moderate risk use	241 (6.7)	176 (73.0)	65 (27.0)
Missing	133 (3.8)	112 (84.2)	21 (15.8)
Smoking			
Nonsmoker	2635 (75.1)	2063 (78.3)	572 (21.7)
Current	875 (24.9)	674 (77.0)	201 (23.0)
Leisure-time physical activity^b^			
High or moderate	2977 (84.8)	2318 (77.9)	659 (22.1)
Low	533 (15.2)	419 (78.6)	114 (21.4)
BMI^c^			
Healthy weight	2039 (58.1)	1681 (82.4)	358 (17.6)
Overweight/obesity	1471 (41.9)	1056 (71.8)	415 (28.2)
Psychological distress^d^			
None	2872 (81.8)	2231 (77.7)	641 (22.3)
Psychological distress	638 (18.2)	506 (79.3)	132 (20.7)
Total	3510 (100)	2737 (78.0)	773 (22.0)

a Alcohol consumption according to Finnish current care guidelines: Women consuming alcohol >7 portions/wk and men consuming >14 portions/wk were considered as moderate risk-users and those consuming less than these as nonrisk-users.^35^

b Leisure-time and commuting physical activity (LTPA): less than 20 metabolic equivalent (MET) hours per week indicates low LTPA and at least 20 MET-hours per week indicates moderate or high LTPA.

c Body mass index (BMI): healthy weight 18.5–24.9 kg/m^2^, overweight/obesity ≥25 kg/m^2^.

d Psychological distress: score of 60 points or less indicates psychological distress and score of over 60 points indicates no psychological distress. Measured with the RAND-36 questionnaire.

**TABLE 2.** Median Values and Interquartile Range (IQR) of Presenteeism Classified by Participants With and Without Psychological Distress (Emotional Well-Being Score ≤60), and With and Without Overweight/Obesity (BMI ≥25 kg/m^2^) Among Young and Early Midlife Participants of the Helsinki Health Study (n = 3510), Phase 1 in 2017

		n (%)	Presenteeism	
			Days/Person/Follow-up Period	
			Median	IQR
Psychological distress				
	No	2872 (81.8)	3.6	11.2
	Yes	638 (18.2)	19.0	18.6
Body mass index				
	Healthy weight	2039 (58.1)	6.9	11.3
	Overweight/obesity	1471 (41.9)	10.4	18.5
Psychological distress and overweight/obesity jointly				
	Neither	1697 (48.3)	0	11.1
	Psychological distress only	342 (9.7)	13.9	16.5
	Overweight/obesity only	1175 (33.5)	8.4	11.3
	Both	296 (8.4)	20.4	21.0

**TABLE 3.** Incidence Rate Ratios (IRRs) and Their 95% Confidence Intervals (CIs) for Presenteeism Days During a 1-Year Follow-up Among Young and Early Midlife Participants of the Helsinki Health Study (n = 3510) With and Without Psychological Distress (Emotional Well-Being Score ≤60), and With and Without Overweight/Obesity (Body Mass Index ≥25 kg/m^2^) at Phase 1 in 2017

Presenteeism Days			Model 1		Model 2		Model 3	
	Joint Exposure Group	n (%)	IRR	95% CI	IRR	95% CI	IRR	95% CI
	Psychological distress and overweight/obesity jointly							
	Neither	1697 (48.3)	1		1		1	
	Pscyhological distress only	342 (9.7)	2.56	2.10–3.13	2.56	2.10–3.13	2.56	2.10–3.12
	Overweight/obesity only	1175 (33.5)	1.14	1.00–1.30	1.11	0.98–1.27	1.10	0.97–1.26
	Both	296 (8.4)	2.91	2.36–3.60	2.85	2.31–3.53	2.80	2.27–3.47

Model 1: Adjusted for gender and age.

Model 2: Adjusted for gender, age, marital status, education, and physical strenuousness of work.

Model 3: Adjusted for gender, age, marital status, education, physical strenuousness of work, alcohol consumption, smoking, and leisure-time physical activity.

Negative binominal regression.

**FIGURE 2.**
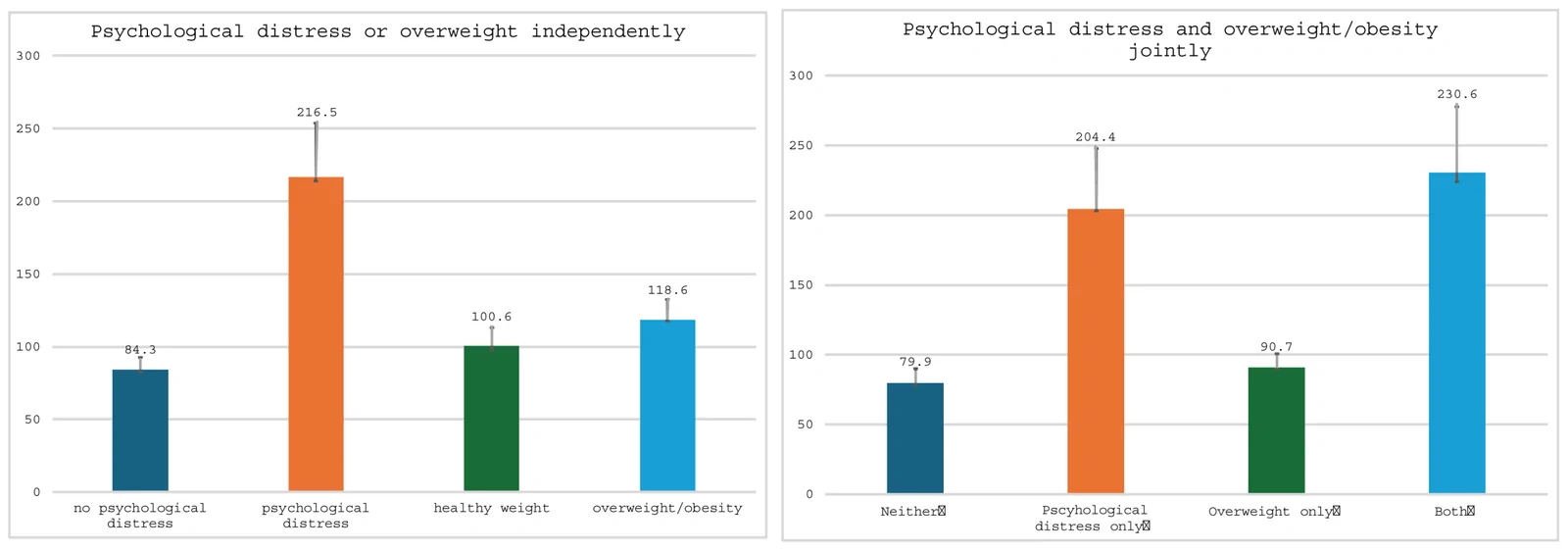
Incidence of presenteeism days during a 1-year follow-up with 95% confidence intervals per 10 person-years according to participants with and without psychological distress (emotional well-being score ≤ 60), and with and without overweight/obesity (body mass index ≥ 25 kg/m^2^) independently and jointly among young and early midlife participants of the Helsinki Health Study (n = 3510), phase 1 in 2017.

As a supplementary analysis, we also considered different cutoff points for psychological distress and BMI, but the results remained essentially similar. We categorized participants into those with a RAND-36 emotional wellbeing score >68 points and those with ≤68 points,^13^ as well as into those with a healthy weight/overweight (18.5–29.9 kg/m^2^) and those with obesity (≥30 kg/m^2^) (Supplemental Digital Content 1, https://links.lww.com/JOM/C567). For comparison to presenteeism days, we presented sickness absence days median and interquartile ranges according to joint exposure groups as a supplementary analysis (Supplemental Digital Content 2, https://links.lww.com/JOM/C568). In Supplemental Digital Content 3, https://links.lww.com/JOM/C569 we presented psychological distress and BMI category independently and by joint exposure groups by sickness absence days per 10 person-years. Gender stratified analyses of the IRRs are presented in Supplemental Digital Content 4, https://links.lww.com/JOM/C570.

We used IBM SPSS Statistics 29 (IBM Corp., Armonk, NY) for all the analyses. This manuscript was prepared using the Strengthening the Reporting of Observational Studies in Epidemiology (STROBE) guidelines (see STROBE checklist, Supplemental Digital Content 5, https://links.lww.com/JOM/C571).

### Ethical Considerations and Disclosure

Appropriate ethical aspects have been followed in all phases of the study, according to the Declaration of Helsinki. Participation in the study was voluntary and full confidentiality was guaranteed. An informed consent to link survey and register data were obtained from each participant. The Helsinki Health Study protocol was approved by the Ethics Committee of the Faculty of Medicine, University of Helsinki, and the City of Helsinki health and personnel authorities. The Faculty of Medicine gave a positive statement for the study protocol (February 14, 2017), and because the study is observational, no other ethical approval was required. The City of Helsinki updated their approval for the Helsinki Health Study protocol on June 21, 2023. https://www.helsinki.fi/en/researchgroups/helsinki-health-study.

## RESULTS

For all participants, presenteeism accounted for 4.4% of total working days, and the majority (58.9%) had at least 1 presenteeism day during the follow-up. Descriptive Table 1 shows cross-tabulation of different variables by gender. Among the participants, 18.2% had psychological distress and 41.9% had overweight/obesity. Table 2 shows median values of presenteeism days within joint exposure groups during the follow-up period. The highest number of presenteeism days was observed among participants with both psychological distress and overweight/obesity, with a median of 20.4 days. As well, those with either psychological distress or overweight/obesity only had more presenteeism days compared with those without psychological distress and having a healthy weight.

Figure 2 presents presenteeism days per 10 person-years by psychological distress, BMI, and joint exposure groups. The highest number of presenteeism days per 10 person-years was observed in the group with both psychological distress and overweight/obesity: 230.6 presenteeism days per 10 person-years. When examined separately, participants with psychological distress had more presenteeism days than those without, and those with overweight had more than those with healthy weight.

In the regression analysis, participants with 1 or both exposures had higher IRRs for presenteeism days over the follow-up compared with those without psychological distress and with a healthy weight (Table 3). The strongest association was observed in the group with both psychological distress and overweight/obesity (IRR: 2.91, CI 2.36–3.60). The association was also strong for participants with psychological distress and a healthy weight (IRR: 2.56, CI 2.10–3.13), and moderate for those without psychological distress and with overweight/obesity (IRR: 1.14, CI 1.00–1.30). Adjustments for marital status, education, and physical strenuousness of work (Model 2), as well as full adjustment including health behaviors (alcohol consumption, smoking, and leisure-time physical activity) (Model 3), only slightly attenuated the associations. Additionally, we calculated IRRs and their 95% CIs for presenteeism days by joint exposure groups separately for women and men, and the results remained the same (Supplemental Digital Content 4, https://links.lww.com/JOM/C570). The interaction of psychological distress and overweight/obesity was additive for presenteeism days in Model 1 (*S* = 1.12) and Model 3 (*S* = 1.08).

## DISCUSSION

Our study demonstrates that especially young and early midlife employees with both psychological distress and overweight/obesity were associated with increased presenteeism. Psychological distress was also independently associated with increased association with. Our results showed weak independent associations between overweight/obesity and presenteeism.

To the best of our knowledge, no previous study has examined the joint contribution of psychological distress and overweight/obesity to presenteeism; prior studies have assessed these factors separately. Their joint association with higher presenteeism may reflect the combined burden of coexisting physical and mental health conditions. The relation between psychological distress and overweight/obesity is complex and bidirectional.^12,40^ The joint association of psychological distress and overweight/obesity with presenteeism was stronger than that of psychological distress alone, with overweight/obesity contributing to the observed association. Employees with both psychological distress and overweight/obesity may have greater need for work recovery. Difficulty balancing work demands and recovery needs has previously been associated with sickness absence.^41^ A similar phenomenon may be observed with presenteeism as well.

We found a strong independent association between psychological distress and presenteeism, consistent with findings from previous studies.^5,15^ Presenteeism and sickness absence reflect work attendance decisions, with subjective health perception playing a central role. Psychological distress-related factors such as job insecurity, low job satisfaction, reduced work engagement, and emotional exhaustion may also influence this choice.^1^ In Finland, mental disorders are a leading cause of sickness absence among young employees, highlighting the need to address presenteeism as a potential early indicator of declining mental health and work ability.^8,10^ Higher psychological distress may reflect reduced functioning not yet leading to sickness absence, and presenteeism may occur both before and after sickness absence periods.^8^

In this study, we found a weak association between overweight/obesity and presenteeism, a finding that contrasts with the inconclusive evidence from previous research.^18,19^ In a comparable occupational setting, a Belgian prospective study of employees aged 30–55 years reported higher overall presenteeism among women, although the association between overweight and presenteeism was observed only among men.^19^ As comparison, Supplemental Digital Content 1, https://links.lww.com/JOM/C567 shows an association between obesity and presenteeism. Notably, in our study, the prevalence of overweight/obesity was slightly lower than nationally in year 2017 in Finland in the age group of 30–39 years, (men 53.7% vs. 61.9% and women 38.6% vs. 44.2%).^42^ This may be explained by the fact that our participants were employed, while higher body weight is more common among individuals outside the workforce.^43^

It is important to note the presenteeism rate in this population. Comparisons across countries should be made with caution owing to differences in social security systems and sickness absence policies, which may influence presenteeism behavior. Age is also a key factor affecting health and presenteeism/sickness absence; thus, findings from older employees may not directly generalize to younger cohorts.^44^ Nearly 60% of participants had at least 1 day of presenteeism during the follow-up, a proportion comparable to that reported in previous studies on presenteeism,^1,19,45^ highlighting the commonality of this phenomenon among young and early midlife municipal employees. In our study, most participants were women (80%). Previous research on gender differences in presenteeism is inconsistent. Some studies report higher presenteeism among women and younger employee,^1,19^ whereas our study found no significant gender differences. Similarly, a Swedish cross-sectional study of care workers found no gender differences in presenteeism after adjustment for education, heavy physical work, and health status.^46^

When comparing presenteeism days to sickness absence days, in most exposure groups, the median number of presenteeism days exceeded that of sickness absence days (Supplemental Digital Content 2, https://links.lww.com/JOM/C568). Additionally, the independent and joint associations of psychological distress and BMI with sickness absence were highly similar to their associations with presenteeism (Supplemental Digital Content 3, https://links.lww.com/JOM/C569). Overall, productivity losses may be particularly high when psychological distress and overweight/obesity contribute jointly to both presenteeism and sickness absence. Our findings suggest that presenteeism may be as important for productivity loss as sickness absence.

There is no golden standard or consensus estimating productivity loss when evaluating presenteeism.^3^ However, in healthcare decision-making, estimating presenteeism are often based on self-reported assessments, and it is unknown whether the measures of productivity loss are comparable.^3^ Even a coarse estimate of health-related productivity loss may help identify risk groups and guide targeted support. Assessing presenteeism is important, as the resulting productivity loss is fully borne by the employer. Addressing presenteeism among employees with long working careers ahead requires a multichannel approach, including workplace health promotion, return-to-work support, and attention to workplace culture.^5^

### Methodological Considerations

#### Limitations and Strengths

The limitations of this study need to be considered. First, psychological distress, BMI, and all the covariates were self-reported. Nevertheless, our previous research has confirmed that self-reported BMI has predictive value for sickness absence and is comparable to the measured BMI.^47^ However, self-reported values in BMI tend to be somewhat underestimated.^47^ Secondly, the questionnaire did not directly assess pregnancy, which may have resulted in inclusion of some pregnancy-related weight gain. Although participants reporting pregnancy elsewhere in the questionnaire were excluded (n = 67), lack of systematic baseline assessment remains a limitation. Thirdly, according to the nonresponse analysis, nonrespondents were more often men, had lower incomes, worked part-time, were manual workers, and had more sickness absence periods.^27^ Nevertheless, the differences in socioeconomic, workplace, and health-related factors between respondents to the online, mail, and phone surveys were minor, suggesting that the data adequately represented the target population. ^27^ Overall, the study sample can be considered representative of young and early midlife municipal employees in Finland, as most municipal workers are women and the response rate was satisfactory.

Fourthly, the method used to calculate presenteeism days has not been validated in the public sector. Presenteeism was assessed using an indirect measurement tool developed and validated by Vänni et al.^28^ The tool is grounded in the WAS, and its validity has been demonstrated in 2 Finnish industrial sectors: the food and forest industries.^32^ To the best of our knowledge, no comparable WAS-based method is currently in use. Because presenteeism is influenced by multiple factors beyond health status, any current assessment can only be regarded as an estimate based on the best available evidence. WAS is influenced by both physical and psychological/mental health and it is strongly associated with overall health status.^22^ The holistic image of work ability, “Work Ability House,” is a conceptual model illustrating work ability as the dynamic balance between individual resources (health, competence, values, and motivation) and work-related factors within the organizational and societal context.^22^ Thus, it can be considered that the presenteeism tool reflects the conditions under in which it is measured reasonably well. “Adjustment for work-related factors was limited to physical strenuousness; occupation, contract type, and psychosocial working conditions were excluded from the main models to avoid overadjustment.”

A major strength of this study is the large cohort representing a wide range of different occupations in the public sector. Secondly, we could utilize objective register data on employer verified sickness absence and length of employment during the follow-up period, both of which were essential for calculating presenteeism days. This helped to avoid self-report bias and nonresponse related to sickness absence. Thirdly, presenteeism is more common in occupations that require physical presence or where arranging a substitute is challenging.^2^ As the typical sectors in the City of Helsinki include healthcare, social work, and education, it is well justified to study presenteeism among this population group.

## CONCLUSION

Among young and early midlife Finnish municipal employees, psychological distress and overweight/obesity were associated with a higher number of presenteeism days. Presenteeism reflects work attendance decisions shaped not only by health status, but also by job demands, work engagement, and psychosocial factors. Addressing these conditions together is important, as treating 1 without the other may limit improvements in health and well-being.^12^ Psychological distress combined with overweight/obesity contributes to productivity loss through increased presenteeism, highlighting the need to address underlying factors and support both mental and physical health in collaboration with workplaces and occupational healthcare services.

## ACKNOWLEDGMENTS

We thank the City of Helsinki, Finland, and all the participants of this study.

## Supplementary Material


