# Supplementary material for: Joint Contributions of Psychological Distress and Body Mass Index to Presenteeism: A Register-linked Follow-up Study Among Young and Early Midlife Municipal Employees
Source: J Occup Environ Med. 2026 Jul 1;68(11):868–74. doi: 10.1097/JOM.0000000000003800 (PMC13686625; doi:10.1097/JOM.0000000000003800)
Supplement: Supplementary file 1 [file joem-68-868-s001.pdf]

Supplemental Digital Content 1. Incidence rate ratios (IRRs) and their 95% confidence intervals (CIs) for presenteeism days during a 1-year follow-up among young and early midlife participants of the Helsinki Health Study (n = 3,510) with and without psychological distress (emotional wellbeing score < 68), and with and without exposure to obesity (BMI ≥ 30 kg/m^2), Phase 1 in 2017. Negative binominal regression.

Presenteeism days			Model 1		Model 2		Model 3	
	Joint exposure group	n (%)	IRR	95% CI	IRR	95% CI	IRR	95% CI
	Psychological distress and obesity							
	Neither	2164	1		1		1	
	Pscyhological distress only	817	2.65	2.31–3.03	2.64	2.30–3.03	2.65	2.31–3.04
	Obesity only	341	1.36	1.12–1.65	1.33	1.09–1.61	1.31	1.07–1.59
	Both	188	2.89	2.25–3.72	2.86	2.22–3.68	2.80	2.17–3.61

Model 1: Adjusted for gender and age

Model 2: Adjusted for gender, age, marital status, education, and physical strenuousness of work

Model 3: Adjusted for gender, age, marital status, education, physical strenuousness of work, alcohol consumption, smoking, and leisure-time physical activity
