# Supplementary material for: Joint Contributions of Psychological Distress and Body Mass Index to Presenteeism: A Register-linked Follow-up Study Among Young and Early Midlife Municipal Employees
Source: J Occup Environ Med. 2026 Jul 1;68(11):868–74. doi: 10.1097/JOM.0000000000003800 (PMC13686625; doi:10.1097/JOM.0000000000003800)
Supplement: Supplementary file 2 [file joem-68-868-s002.pdf]

Supplemental Digital Content 2. Median values and interquartile range (IQR) of sickness absence classified by participants with and without psychological distress (emotional wellbeing score ≤ 60), and with and without overweight/obesity (BMI ≥ 25 kg/m^2) among young and early midlife participants of the Helsinki Health Study (n = 3,510), Phase 1 in 2017.

			Sickness absence Days/person/follow-up period	
		n (%)	Median	IQR
Psychological distress				
	No	2,872 (81.8)	5.0	11.0
	Yes	638 (18.2)	8.0	15.0
Body mass index				
	Healthy weight	2,039 (58.1)	4.0	10.0
	Overweight/obesity	1,471 (41.9)	6.0	13.0
Psychological distress and overweight/obesity				
	Neither	1,697 (48.3)	4.0	10.0
	Pscyhological distress only	342 (9.7)	7.0	13.0
	Overweight/obesity only	1,175 (33.5)	6.0	12.0
	Both	296 (8.2)	8.0	18.0
