# Supplementary material for: Joint Contributions of Psychological Distress and Body Mass Index to Presenteeism: A Register-linked Follow-up Study Among Young and Early Midlife Municipal Employees
Source: J Occup Environ Med. 2026 Jul 1;68(11):868–74. doi: 10.1097/JOM.0000000000003800 (PMC13686625; doi:10.1097/JOM.0000000000003800)

Supplemental Digital Content 3. Incidence of sickness absence days during a 1-year follow-up with 95% confidence intervals per 10 person-years according to participants with and without psychological distress (emotional well-being score ≤ 60), and with and without overweight/obesity (body mass index ≥ 25 kg/m2) independently and jointly among young and early midlife participants of the Helsinki Health Study (n = 3,510), Phase 1 in 2017.

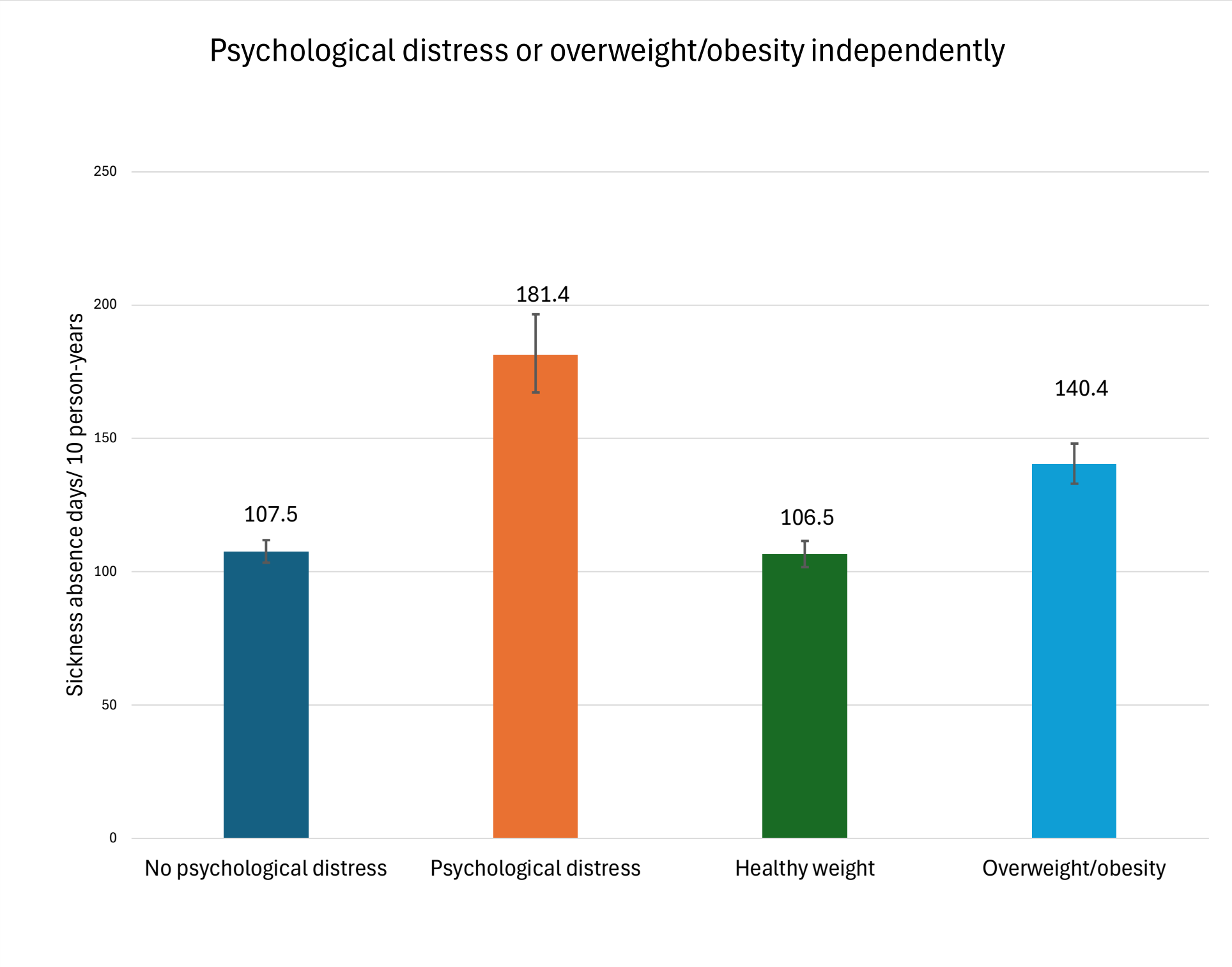

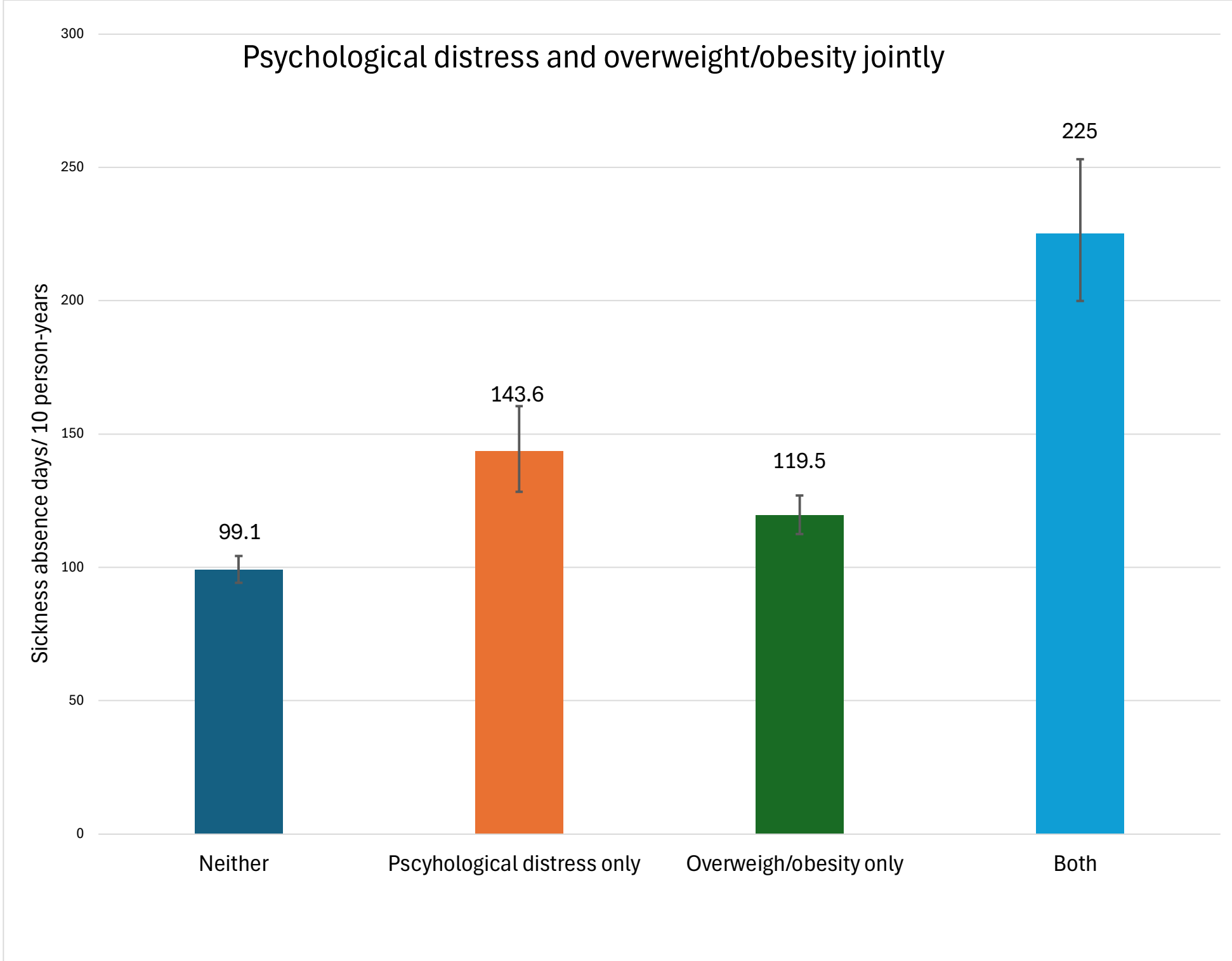

Supplement: Supplementary file 3 [file joem-68-868-s003.pdf]
