# Supplementary material for: Joint Contributions of Psychological Distress and Body Mass Index to Presenteeism: A Register-linked Follow-up Study Among Young and Early Midlife Municipal Employees
Source: J Occup Environ Med. 2026 Jul 1;68(11):868–74. doi: 10.1097/JOM.0000000000003800 (PMC13686625; doi:10.1097/JOM.0000000000003800)
Supplement: Supplementary file 4 [file joem-68-868-s004.pdf]

Supplemental Digital Content 4. Incidence rate ratios (IRRs) and their 95% confidence intervals (CIs) for presenteeism days during a 1-year follow-up among young and early midlife participants of the Helsinki Health Study (n = 3,510) with and without psychological distress (emotional well-being score ≤ 60), and with and without overweight/obesity (body mass index ≥ 25 kg/m^2) at Phase 1 in 2017, gender stratified. Negative binominal regression.

Presenteeism days				Model 1		Model 2		Model 3	
	Joint exposure group		n (%)	IRR	95% CI	IRR	95% CI	IRR	95% CI
Women	Psychological distress and overweight/obesity jointly								
		Neither	1391 (50.8)	1		1		1	
		Pscyhological distress only	290 (10.6)	2.53	2.05–3.12	2.53	2.05–3.12	2.53	2.05–3.12
		Overweight/obesity only	840 (30.7)	1.19	1.03–1.37	1.16	1.01–1.35	1.16	1.00–1.34
		Both	216 (7.9)	2.8	2.21–3.55	2.74	2.16–3.48	2.72	2.14–3.46
Men	Psychological distress and overweight/obesity jointly								
		Neither	306 (39.6)	1		1		1	
		Pscyhological distress only	52 (6.7)	2.73	1.55–4.80	2.71	1.54–4.77	2.72	1.54–4.79
		Overweight/obesity only	335 (43.3)	0.97	0.72–1.32	0.78	0.70–1.30	0.96	0.71–1.31
		Both	80 (10.3)	3.19	1.99–5.12	3.09	1.92–4.99	3.08	1.91–4.97

Model 1: Adjusted for age

Model 2: Adjusted for age, marital status, education, and physical strenuousness of work

Model 3: Adjusted for age, marital status, education, physical strenousness of work, alcohol consumption, smoking, and leisure-time physical activity
